# Ultrasound evaluation of gallbladder wall thickness for predicting severe dengue: a systematic review and meta-analysis

**DOI:** 10.1186/s13089-025-00417-5

**Published:** 2025-02-03

**Authors:** Amirhossein Shahsavand Davoudi, Hamid Harandi, Reza Samiee, Shayan Forghani, Keyhan Mohammadi, Maryam Shafaati

**Affiliations:** 1 School of Medicine, Tehran University of Medical Sciences, Tehran, Iran; 2 Research Center for Antibiotic Stewardship and Antimicrobial Resistance, Infectious Diseases Department, Imam Khomeini Hospital Complex, Tehran University of Medical Sciences, Tehran, Iran; 3 Department of Clinical Pharmacy, School of Pharmacy, Tehran University of Medical Sciences, Tehran, Iran

**Keywords:** Dengue fever, Severe dengue, Gallbladder wall thickening (GBWT), Ultrasound, Risk prediction

## Abstract

**Background:**

The prevalence of dengue fever (DF), a mosquito-borne viral disease, is rising worldwide. Its severe manifestations like thrombocytopenia and plasma leakage are associated with increased mortality. Ultrasound-detected gallbladder wall thickening (GBWT) has been suggested as a potential indicator of the severity of the disease.

**Aims:**

This systematic review and meta-analysis evaluated the predictive value of GBWT in identifying patients at risk for severe dengue.

**Methods:**

Following the PRISMA 2020 guidelines, we conducted a systematic search of Web of Science, PubMed, Embase, and Scopus. Among the inclusion criteria were original studies that assessed GBWT across various dengue severity categories. Then, we performed a meta-analysis using a random effects model and subgroup analyses based on severity criteria to determine the relationship between GBWT and severe dengue.

**Results:**

For the meta-analysis, 19 studies qualified for the inclusion criteria. There was a significant association between GBWT and severe dengue, according to the odds ratio (OR) of 2.35 (95% CI 1.88–2.82, p < 0.001). The subgroup analysis revealed consistent results for thrombocytopenia (OR: 2.65) and plasma leakage (OR: 2.26), among other severity criteria.

**Conclusions:**

A reliable ultrasound indicator, GBWT can help identify patients at risk for severe dengue early on, improving clinical decision-making and patient outcomes. However, the possibility of differential diagnosis requires cautious interpretation.

**Supplementary Information:**

The online version contains supplementary material available at 10.1186/s13089-025-00417-5.

## Introduction

The dengue virus, which causes dengue fever (DF), primarily spreads through Aedes mosquitoes. Over the past several decades, the prevalence of DF has dramatically grown globally [1, 2]. Of all arthropod-borne viral infections, dengue virus, which contains serotypes DENV-1–DENV-4, has the highest disease burden globally and causes a self-limiting febrile illness [3].

DF can manifest in an extensive variety of conditions, ranging from asymptomatic cases to severe fever, with or without warning signs. In 1986, the World Health Organization (WHO) established the inaugural classification for dengue severity, differentiating between non-severe and severe cases. A significant change occurred in 1997, improving the classification criteria. Subsequent changes issued in 2009 and 2011 enhanced dengue diagnostics and management strategies. The initial clinical manifestations of dengue are fever, headache, myalgia, and arthralgia. The WHO identifies seven warning signs: abdominal pain, continuous vomiting, accumulation of fluid, mucosal bleeding, lethargy, liver enlargement, and a rise in hematocrit coupled with a drop in platelets [3, 4]. According to the WHO, severe dengue is characterized by significant plasma leakage, severe hemorrhaging, or severe organ involvement. Thrombocytopenia and plasma leakage are significant indicators of complications. Thrombocytopenia is an immune system response to the dengue virus that raises the risk of bleeding. Increased vascular permeability causes plasma leakage, another sign of severe dengue, which could lead to shock and organ failure [5]. Therefore, the path to severe dengue and its determinants is intricate.

Recent epidemics have shown that the non-specific or undifferentiated clinical findings cause delays in diagnosis and treatment [6]. There is currently no validated clinical test or investigation that can predict patients at risk of developing severe dengue and its associated clinical features, but there are some studies suggesting some probable predicting factors for severe dengue [7, 8]. Early risk stratification may help physicians decide on the intensity of observation and treatment for patients, highlighting the need to refine criteria for early identification of those at risk of severe dengue.

Some studies [9–11] suggest abdominal ultrasonography as an effective tool for predicting severe dengue at an early stage of the illness. The gallbladder wall thickening (GBWT) is a relatively common finding in DF [12]. Recent studies suggest that measuring GBWT can serve as a predictive indicator to assess patients at risk of progressing to the critical phase [10, 11, 13]. Studies show a strong link between GBWT levels above 3 mm and more severe dengue cases. A thickness above 5 mm could help physicians find dengue patients who are at a high risk of going into hypovolemic shock [11].

This systematic review and meta-analysis aimed to assess the effectiveness of GBWT, measured using ultrasound, in predicting the likelihood of severe dengue in patients. The existing literature review indicates the need for more comprehensive studies and meta-analyses to fully investigate this possibility. Our aim was to clarify the predictive role of GBWT in dengue severity to facilitate enhanced treatment decisions and improve patient outcomes.

## Methodology

### Protocol and registration

This systematic review and meta-analysis followed the PRISMA 2020 guidelines for reporting and was conducted based on a protocol registered with PROSPERO (No. CRD42024598379).

### Search strategy and study selection

In this meta-analysis, we used the PRISMA (Preferred Reporting Items for Systematic Reviews and Meta-Analyses) 2020 guidelines [14]. We searched the PubMed, Embase, Scopus, and Web of Science databases on August 2, 2024. We selected keywords related to (“Severe Dengue”) AND (“gallbladder” OR “wall thickening”) and the related medical subject headings (MeSH) terms to develop our search strategy (Supplementary file, Table S1). The study involved exporting titles and abstracts into EndNote v.21 software, screening based on titles and abstracts, and full-text screening. The screening process was conducted independently by two authors, with disagreements resolved by a senior author. Additionally, a manual review search of related articles was conducted to identify potential studies.

### Inclusion and exclusion criteria

We incorporated all the original English studies that evaluated the severity of gallbladder wall thickening in at least two groups of dengue patients and included studies that classified dengue severity using WHO classification tools, thrombocytopenia, or plasma leakage. The exclusion criteria were as follows: (1) studies that did not report dengue severity; (2) studies that did not report gallbladder wall thickening; (3) case reports and case series with fewer than five cases; (4) non-original studies (e.g., reviews or commentaries); (5) abstracts or studies without full text; (6) studies with a duplicate database; (7) non-human studies; and (8) non-English studies.

### Data extraction

Before starting the data extraction process, we designed an Excel sheet. Three authors independently extracted the data from the included studies and put it into an Excel sheet. After rechecking its validity, we used the extracted data to synthesize the systematic review and meta-analysis. We included the following information in the data extraction Excel sheet: Study ID, Study design, Study country, Follow-up period, Dengue diagnostic criteria, Mean age of participants with dengue, Male percentage of participants, Imaging modality, Criteria of gallbladder wall thickening, A summary of study conclusions, Dengue severity criteria, each severity classification includes the total number of participants, the number of participants with gallbladder wall thickening, and the number of participants without gallbladder wall thickening (Supplementary file, Table S2). We also construct contingency figures (true positives, false positives, true negatives, and false negatives) for each study and subsequently calculated sensitivity, specificity, positive likelihood ratio (PLR), negative likelihood ratio (NLR), and diagnostic odds ratio (DOR) (Supplementary Figs. 6 to 11).

### Ultrasound protocols for GBWT examination

The studies evaluated gallbladder wall thickening (GBWT) using various ultrasonography procedures. Qualified radiologists or sonographers performed transabdominal ultrasonography in the majority of studies. To minimize variability related to gallbladder contraction or postprandial thickening, some investigations followed established protocols, such as a 4- to 6-h fast before imaging [15, 16]. In both longitudinal and transverse orientations, measurements were typically taken at the gallbladder wall's most reliant region. Most of the time, a thickness of 3 mm or more is used to identify GBWT. However, some studies have looked at using higher thresholds (like 5 mm) or unique patterns (like the "honeycomb" appearance) to make the diagnosis more accurate. Methodological heterogeneity may account for the observed variability, highlighting the need for imaging procedure standardization in future studies.

### Statistical analysis, quality assessment, and publication bias

We conducted a meta-analysis using Stata version 17. We compared the presence of gallbladder wall thickening in the most severe cases of dengue with those with milder severity levels. The severe groups consist of patients with dengue shock syndrome (DSS) in 1986, 1997, and 2011 WHO criteria, and severe dengue (SD) in 2009 WHO criteria. Furthermore, according to WHO guidelines [17], patients with plasma leakage and thrombocytopenia (platelet counts below 100,000) were considered to have severe dengue. We employed a random-effects model and log odds ratio (OD) to assess the relationship between two distinct binary variables. Random-effect models are a better choice to address the high heterogeneity between studies, with an I^2^ value exceeding 40%. Next, we conducted a subgroup analysis using various dengue severity classification tools. For diagnostic accuracy meta-analysis, we employed the MIDAS package in Stata to conduct bivariate random-effects modeling. Pooled diagnostic accuracy parameters were calculated with corresponding 95% confidence intervals (CIs), and the results were presented using a summary receiver operating characteristic (SROC) curve (Table 1). We also assessed the heterogeneity of the included studies using a Galbraith plot and also designed a funnel plot to illustrate the publication bias among the included studies. To assess the quality of the included studies, we used the Newcastle–Ottawa scale (NOS) and the NOS modified version for cross-sectional studies [18, 19].

**Table 1:** Diagnostic accuracy of gallbladder wall thickening for predicting severe dengue

Parameter	Estimation	95% confidence interval (95% CI)
Sensitivity	0.88	[0.77, 0.94]
Specificity	0.63	[0.48, 0.76]
Positive likelihood ratio	2.4	[1.7, 3.3]
Negative likelihood ratio	0.19	[0.11, 0.33]
Diagnostic odds ratio	12	[7, 21]

## Results

### Study selection

**Fig. 1:**
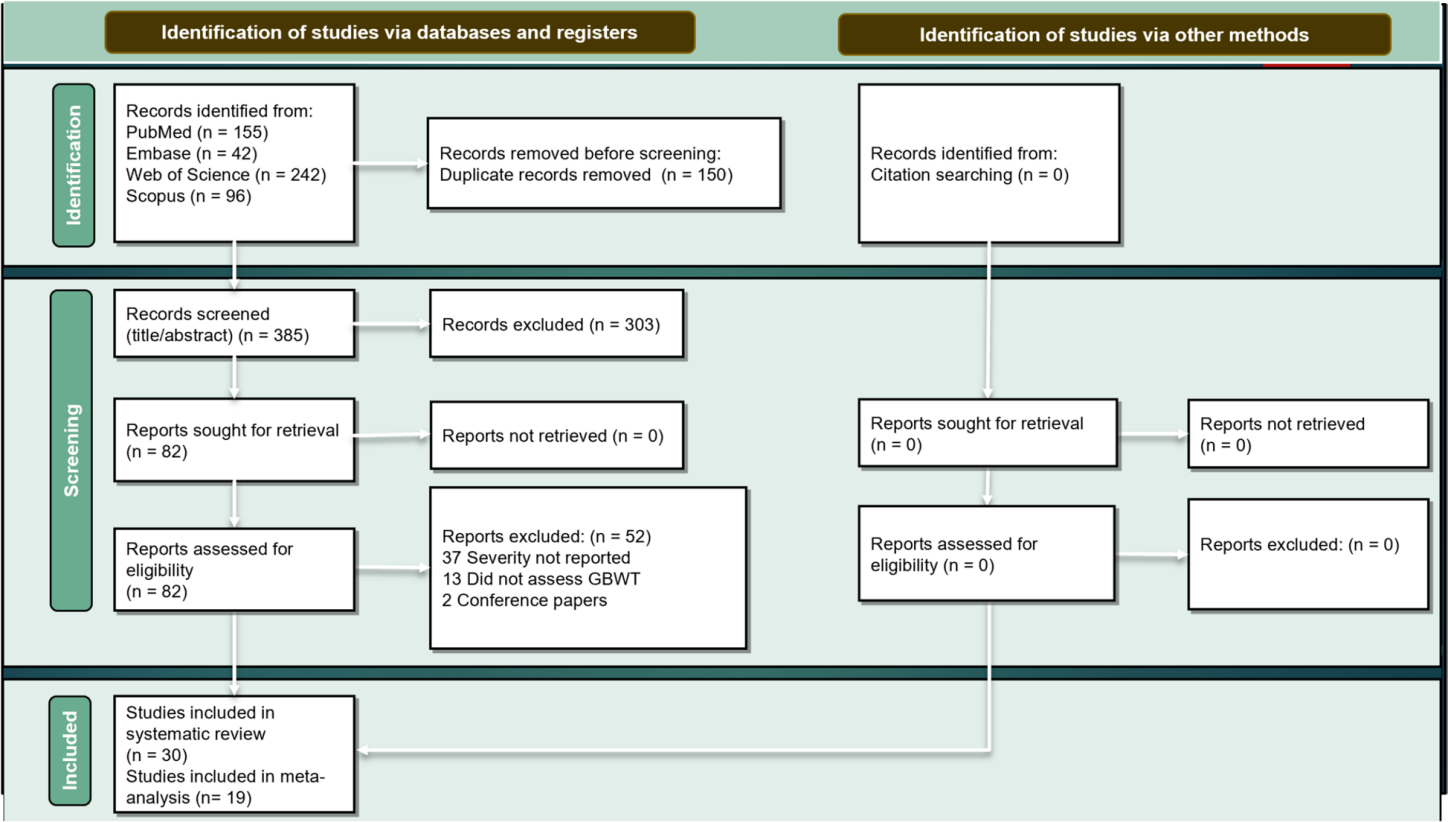
PRISMA flow diagram process and the systematic review methodology

The PRISMA flow diagram, presented in Fig. 1, illustrates the comprehensive search strategy employed in this study. According to the Fig. 1, this systematic approach resulted in the identification of 535 publications following the initial search. After removing duplicates, a total of 385 publications remained. These publications underwent a screening process, with two independent authors evaluating titles and abstracts. The initial title/abstract screening determined the irrelevance of 303 studies to the research question, leading to their exclusion at this stage. The remaining 82 studies underwent a thorough full-text review for eligibility criteria, which led to the exclusion of an additional 52 articles. Ultimately, this rigorous process produced 30 publications that satisfied all inclusion criteria, and this meta-analysis included 19 of them.

### Study characteristic

Table 2 presents a summary of the characteristics of the studies included in this meta-analysis. This systematic review included 30 studies that met the predetermined inclusion criteria. They published the included studies between 1995 and 2024. Asian and South American countries conducted the majority of the included studies. The designs of all of the included studies were cross-sectional and cohort. Most of the studies used serology testing and NS1 antigen detection as their diagnostic criteria for dengue (10–13, 16, 20–42). Some studies [10, 11, 26, 38, 39, 43] utilized real-time PCR and virus isolation. One study [44] did not report the tests used for dengue diagnosis. In Table 3, the different ways that GBWT severity is defined. It also shows all the criteria that are used to classify dengue severity, such as the WHO criteria, thrombocytopenia, bleeding episodes, severe plasma leakage, and patient outcome, which are shown in Table 2. Table 3 provides a critical reference for understanding the criteria used. In studies aimed at classifying dengue severity, it's crucial to ensure consistency in the synthesis of data and the interpretation of findings in meta-analyses.

**Table 2:** Studies characteristics provides a comprehensive summary of the key details from the studies included in the systematic review and meta-analysis

References	Study design	Country	Study period	Dengue diagnostic criteria	Dengue severity criteria	Mean age ± SD	Sex, males	Number of participants	Definition of GBWT	Assesment of GBWT
Adil et al. [20]	Cohort	Pakistan	September 2019–January 2020	NS1 antigen Serology (IgM)	WHO 1997	33 ± 13	67.80%	DF: 106	GBWT above 3mm	Two experienced radiologists
								DHS: 68		
								DSS: 6		
Agarwal and P. Jain [21]	Cohort	India	June 2015–November 2015	Serology (IgM)	Platelet count	N/R	N/R	PLT > 100000: 19	GBWT above 3mm	N/R
								PLT 50000–100000: 49		
								PLT < 50000: 58		
Asghar et al. [22]	Cross-sectional	Pakistan	N/R	NS1 antigen Serology (IgM)	Platelet count and bleeding events	33.2 ± 13.6	54.80%	Total: 177	GBWT above 3mm	N/R
Bandyopadhyay et al. [23]	Cross-sectional	India	July 2014–June 2015	NS1 antigen Serology (IgM)	WHO 1997	N/R	55.50%	DF: 59	N/R	N/R
								DHS: 26		
								DSS: 25		
Bharath Kumar Reddy et al. [24]	Cohort	India	September 2010–July 2012	Serology (IgM)	WHO 1997 (only DHF grades comparison)	Children	N/R	DHF 1: 52	GBWT above 3mm	One exprienced radiologist
								DHF 2: 96		
								DHF 3: 132		
								DHF 4: 44		
Binh et al. [43]	Cohort	Vietnam	July 2007–October 2007	RT-PCR virus isolation	Thrombocytopenia	24.8 ± 7.3	57.60%	PLT > 100000: 100	N/R	N/R
								PLT < 100000: 51		
Chacko and G. Subramanian [44]	Cohort	India	September 2005–December 2005	N/R	WHO 1997 DSS and non DSS (DF and DHS)	7.87	60%	DSS: 34	N/R	N/R
								Non DSS: 39		
Chaudhary et al. [25]	Cohort	India	October 2016–December 2017	NS1 antigen Serology (IgM)	WHO 1997	30.5 ± 13.4	68%	DF: 255	GBWT above 3mm	One radiologist > 10 years exprence, and 1 year experience
								DHF: 146		
								DSS: 17		
de Araújo Tavares et al. [26]	Cross-sectional	Brazil	January 2011–May 2011	NS1 antigen RT-PCR	WHO 2009	31	19.50%	DWoW: 0	GBWT above 3mm	One physician
								DWS: 31		
								SD: 87		
Donaldson et al. [27]	Cohort	Sri Lanka	December 2016–August 2018	NS1 antigen Serology (IgM)	WHO 2009	34	48.80%	DWS and DWoW: 106	N/R	Two radiologists with > 10 years experience
								SD: 70		
Sahana et al. [28]	Cohort	India	July 2012–February 2013	NS1 antigen Serology (IgM, IgG)	WHO 2009	8	67.90%	DWoW: 39	N/R	N/R
								DWS: 22		
								SD: 20		
Santhosh et al. [12]	Cross-sectional	India	April 2012–August 2012	NS1 antigen Serology (IgM, IgG)	Thrombocytopenia	26	N/R	PLT > 150000: 5	N/R	One radiologist > 5 years exprence
								PLT 80000–150000: 15		
								PLT 40000–80000: 30		
								PLT < 40000: 46		
Setiawan et al. [11]	Cohort	Indonesia	November 1990–February 1993	Virus isolation Serology	WHO 1986 (with modification for bleeding manifestatin)	Range [5 m-14 y]	N/R	DHF 1,2: 48	GBWT above 3mm	N/R
								DHF 3,4: 48		
Setiawan et al. [10]	Cohort	Indonesia	November 1990–June 1994	HI assay Serology virus isolation	WHO 1986 (with modification for bleeding manifestatio)	Range (5m-14y)	50.70%	DHF 1,2: 73	GBWT above 3mm	N/R
								DHF 3,4: 75		
Tauseef et al. [29]	Cross-sectional	Pakistan	N/R	Serology (IgM)	Outcomes (shock or recovered)	30	76%	Shock: 7	N/R	N/R
								Recovered: 43		
Uthraraj et al. [30]	Cohort	India	3 months	NS1 antigen	Thrombocytopenia and plasma leakage	9.17	N/R	Total: 52	GBWT above 5mm	N/R
Vedaraju et al. [31]	Cross-sectional	India	July 2015–September 2015	NS1 antigen Serology (IgM, IgG)	Thrombocytopenia	27.8	53%	PLT > 150000: 1	N/R	N/R
								PLT 80000–150000: 12		
								PLT 40000–80000: 33		
								PLT < 40000: 54		
Venkata Sai et al. [32]	Cross-sectional	India	June 2002–July 2002	Serology	N/R	5	62.50%	Total: 88	N/R	N/R
Yousaf et al. [33]	Cross-sectional	Pakistan	August 2010–December 2010	Serology	WHO 2009	29	54.40%	DF: 103	GBWT above 3mm	N/R
								DHF: 49		
								DSS: 6		
Zulkarnain et al. [34]	Cross-sectional	Indonesia	January 1997–December 1997	Serology (IgM, IgG)	Hemoconcentration (plasma leakage)	N/R	N/R	Increase HCT < 10%: 29	GBWT above 2mm	N/R
								Increase HCT 10%-20%: 20		
								Increase HCT > 20%: 8		
Ibrahim et al. [35]	Cross-sectional	Malaysia	March–September 2018	NS1 antigen Serology (IgM)	WHO 2009	39 ± 15	N/R	DWS: 23	GBWT above 3mm	N/R
								SD: 21		
Jain et al. [36]	Cross-sectional	India	March 2017–March 2018	NS1 antigen Serology (IgM, IgG)	N/R	N/R	N/R	Mild: 21	GBWT above 3mm	N/R
								Moderate: 23		
								Severe: 11		
Mallhi et al. [37]	Cohort	Malaysia	Jan 2008–Dec 2013	NS1 antigen Serology (IgM, IgG)	WHO 2011 guidline	30.69 ± 16.13	56.70%	DF: 588	N/R	N/R
								DHF: 69		
								DSS: 10		
Michels et al. [38]	Cohort	Indonesia	March 2011–January 2012	RT-PCR Serology (IgM, IgG)	WHO 2009	20.1	57.57%	Non severe: 55	GBWT above 3mm	Exprienced physicians
								Severe: 11		
Nainggolan et al. [39]	Cross-sectional	Indonesia	July 2011–October 2012	RT-PCR NS1 antigen	WHO 1997	24.2 ± 10	52.20%	No plasma leakage: 23	GBWT above 3mm	One experienced sonographer
								Plasma leakage: 46		
Oliveira et al. [40]	Cohort	Brazil	January–April 2009	Serology (IgM)	WHO 1997	8.3	51.35%	Severe dengue: 37	GBWT above 3mm	One radiology resident, and one sonographer with > 5 years experience, reviewed by a radiologist with 37 years experience
Osorio et al. [13]	Cohort	Colombia	April 2019–March 2020	NS1 antigen Serology (IgM, IgG)	WHO 2009	18.2	44.40%	DWoS: 80	GBWT above 3mm	One general physician, and one experienced radiologist
								SD: 98		
Parmar et al. [16]	Cross-sectional	India	July 2015–December 2015	NS1 antigen Serology (IgM, IgG)	WHO 2009	32.28 ± 15.0	64.50%	DWoW: 38	GBWT above 3mm	One radiologist
								DWS: 26		
								SD: 29		
Parmar et al. [41]	Cross-sectional	India	2016	NS1 antigen Serology (IgM, IgG)	WHO 2009	Range (1–81)	59.42%	DWOW: 99	GBWT above 3mm	One radiologist
								DWS: 61		
								SD: 84		
Pothapregada et al. [42]	Cohort	India	August 2012–January 2015	NS1 antigen Serology (IgM, IgG)	Severe plasma leakage	7 ± 3.3	54.50%	Non severe: 159	GBWT above 3mm	One radiologist
								Severe: 95		

*NS1* dengue nonstructural protein-1, *RT-PCR* Reverse transcription polymerase chain reaction, *HI* hemagglutination inhibition assay, *PLT* Platelet Count, *HCT* Hematocrit, *DF* Dengue Fever, *DHF* Dengue Hemorrhagic Fever, *DSS* Dengue Shock Syndrome, *DWoS* Dengue Without Warning Signs, *DWWS* Dengue with Warning Signs, *SD* Severe Dengue, *GBWT* Gallbladder wall thickness, *N/R* Not reported

**Table 3:** WHO severity classification. It provides a detailed overview of the WHO severity classification criteria for dengue across different iterations (1986, 1997, 2009, and 2011)

Severity criteria	Severity group		Group	Definition
1986 WHO criteria	DHF	Mild	Grade 1	Fever accompanied by non-specific constitutional symptoms; the only haemorrhagic manifestation is a positive tourniquet test
			Grade 2	Spontaneous bleeding in addition to the manifestations of Grade I patients, usually in the form of skin and/or other haemorrhages
		Severe (DSS)	Grade 3	Circulatory collapse manifested by rapid and weak pulse, narrowing of pulse pressure (20 mmHg or less) or hypotension, with the presence of cold clammy skin and restlessness
			Grade 4	Profound shock with undetectable blood pressure and pulse
1997 WHO criteria	Mild		DF	An acute illness characterized by fever and at least two or more of the following symptoms: headache, pain behind the eyes, muscle pain, joint pain, skin rash, bleeding manifestations, or a low white blood cell count
			DHF (Grade 1 and 2)	All of the following must be present: fever or a recent history of fever, signs of bleeding (such as a positive tourniquet test, petechiae/purpura/ecchymoses, mucosal bleeding), low platelet count, and plasma leakage indicated by an increase in hematocrit or the presence of pleural effusion or ascites
	Severe		DSS (Grade 3 and 4)	Dengue Shock Syndrome requires all the criteria for DHF in addition to signs of circulatory collapse such as a rapid, weak and narrow pulse (less than 20 mm Hg) and hypotention with the presence of cold clammy skin and restlessness
2009 WHO criteria	Mild		DWoWS	A fever accompanied by two or more of the following symptoms: nausea or vomiting, a rash, body aches, a positive tourniquet test, or a low white blood cell count
			DWWS	Similar to DWoWS, but with additional warning signs, suach as abdominal pain, persistent vomiting, lethargy or restlessness, liver enlargement, an increase in hematocrit, and a drop in platelet count
	Severe		SD	Severe dengue is identified by one or more of the following: severe plasma leakage causing shock, fluid overload leading to respiratory distress, severe bleeding, or serious organ dysfunction
2011 WHO guideline	DF			Fever with two of the following: Headache, Retro-orbital pain, Myalgia, Arthtralgia/bone pain, Rash, Haemorrhagic Manifestations, No evidence of plasma leakage. Leucopenia (wbc ≤ 5000cells/mm^3^). Thrombocytopenia (Platelet Count < 150 000 cells/mm^3^), Rising haematocrit (5–10%), No evidence of plasma loss
	DHF		Grade 1	Fever and haemorrhagic manifestation (positive tourniquet test) and evidence of plasma leakage and laboratory finding like Thrombocytopenia < 100 000 cells/mm^3^ and HCT rise ≥ 20%
			Grade 2	Similar to Grade I plus spontaneous bleeding and laboratory finding like Thrombocytopenia < 100 000 cells/mm^3^ and HCT rise ≥ 20%
	DSS		Grade 3	Similar to Grade I or II plus circulatory failure (weak pulse, narrow pulse pressure (≤ 20 mmHg), hypotension, restlessness) and laboratory finding like Thrombocytopenia < 100 000 cells/mm^3^ and HCT rise ≥ 20%
			Grade 4	Similar to Grade III, plus profound shock with undetectable Blood Pressure and laboratory finding like Thrombocytopenia < 100 000 cells/mm^3^ and HCT rise ≥ 20%

Each classification outlines the clinical and laboratory features used to distinguish between mild, moderate, and severe forms of the disease, with specific emphasis on parameters such as plasma leakage, thrombocytopenia, and organ involvement

*DF* Dengue Fever, *DHF* Dengue Hemorrhagic Fever, *DSS* Dengue Shock Syndrome, *DWoS* Dengue Without Warning Signs, *DWWS* Dengue with Warning Signs, *SD* Severe Dengue, *HCT* Hematocrit

### Quality assessment and publication bias

Supplementary files, Tables S3 and S4 also displays the results of the quality assessment of the included studies. The quality of the included studies was evaluated using the Newcastle–Ottawa Scale (NOS) for cohort studies (Table S3) and a modified version of the NOS for cross-sectional studies (Table S4).

### Meta-analysis

In Supplementary Fig. 2 demonstrates that 19 study arms within the included studies explored the correlation between gallbladder wall thickening and disease severity. A significant association was observed between increased GBWT and severe dengue infections, with an odds ratio (OR) of 2.35 (95% CI 1.88–2.82, p < 0.001, I^2^ = 58.90%).

Additionally, a subgroup analysis focused on alternative severity markers employed in the included studies. The subgroup analyses using alternative dengue severity criteria yielded results consistent with the overall analysis. For thrombocytopenia, the OR was 2.65 (95% CI 1.66–3.64). The odds ratio for the WHO 1997 criteria was 1.87 (95% CI 0.77–2.97), for the WHO 2009 criteria 2.29 (95% CI 1.60–2.98), and for plasma leakage 2.26 (95% CI 0.54–3.99). The single study employing the WHO 1986 criteria reported an OR of 3.65 (95% CI 2.53–4.77) (Supplementary Fig. 3).

All subgroups demonstrated a statistically significant difference in GBWT between severe and non-severe groups, as summarized in Table 4.

**Table 4:** Subgroup analysis results to explore the association between gallbladder wall thickening (GBWT) and severe dengue based on various severity classification criteria

Subgroup	Number of studies	Severe dengue		Mild dengue		Test of teta		Heterogeneity		
		GBWT +	GBWT −	GBWT +	GBWT –−	Z	P	T^2^	I^2^	H^2^
WHO 1997	4	196	28	327	481	3.33	< 0.01	0.70	63.56%	2.74
WHO 1986	1	71	4	23	50	6.38	< 0.01	–	–	
WHO 2009	8	230	98	313	339	6.47	< 0.01	0.43	47.63%	1.91
Thrombocytopenia	4	174	35	107	157	5.23	< 0.01	0.43	42.03%	1.73
Plasma leakage	2	53	88	9	173	2.57	0.01	1.13	72.29%	3.61

*GBWT* Gallbladder wall thickness

Subgroup: The specific severity criteria or classification used (e.g., WHO 1997, WHO 2009, thrombocytopenia, plasma leakage)

Number of studies: The total number of studies contributing data to each subgroup

GBWT + and GBWT − : Number of patients with and without gallbladder wall thickening in both severe and mild dengue groups

Statistical results: Odds ratios (OR), confidence intervals (CI), and significance values (e.g., Z and P-values) highlighting the strength of association

Heterogeneity indicators: Metrics such as Tau^2^, I^2^, and H^2^, indicating the variability across studies

Here, we employed a visual assessment of the funnel plot to evaluate the extent of publication bias. The funnel plot revealed no evidence of publication bias concerning the outcome, as shown in Supplementary Fig. 4. The Galbraith plot for the heterogeneity assessment indicated the presence of only minor outlier studies (Supplementary Fig. 5).

## Discussion

In this systematic review and meta-analysis, we found that patients with severe dengue disease have higher gallbladder wall thickening (GBWT) compared to patients with non-severe dengue. In the setting of confirmed dengue patients without any comorbidity, GBWT has estimated sensitivity and specificity of 88 and 63%, respectively. These findings underscore the association between GBWT and dengue severity. We also found that the severity of plasma leakage and thrombocytopenia is associated with GBWT. Therefore, GBWT could serve as a potential severity indicator and a reliable predictive marker for better classification and management of dengue cases.

Dengue fever (DF) is an acute, febrile illness that is typically self-limiting, with a mortality rate of less than 1% [45]; however, some patients progress into severe dengue manifestations, including dengue hemorrhagic fever (DHF) and dengue shock syndrome (DSS). Severe dengue has a higher mortality rate; without treatment, severe dengue can lead to mortality in 10–20% of patients [46, 47], but early detection and proper management can reduce the mortality rate to 2–5% [48]. Diagnosis of DF relies on clinical findings, laboratory tests, and serological examinations [49]. The rapid progression of dengue can potentially outpace many laboratory tests [33, 41], this underscores the importance of employing sensitive diagnostic tools and practical guidelines for identification and severity-categorization of dengue patients, especially in emergency settings [41, 43].

Management of severe dengue critically depends on the early detection of warning signs before the onset of shock [50]. Patients with predisposing factors such as older or younger age [51, 52], higher weight [53], female sex, white ethnicity [52], secondary dengue infection [51, 52, 54], and underlying comorbidities like cardiovascular disease, diabetes, renal disease, hypertension, and pulmonary disease [51, 52, 54], are more prone to develop severe dengue. Clinical findings associated with severe dengue includes abdominal pain, lethargy, nausea and vomiting, headache, myalgia or arthralgia [51, 54]. Early indicators of severe dengue include laboratory findings such as elevated c-reactive protein (CRP), elevated aspartate aminotransferase (AST) [55], decreased serum albumin, and decreased platelet count [52, 55]. Imaging findings, such as pleural effusion and ascites, are also significant markers of severity [50, 52]. The association between increased hematocrit levels and severe dengue remains controversial, with varying evidence on its predictive value [52, 54]. GBWT is suggested to “precede the clinical detection” of severe dengue [50], however, the utility of GBWT in early prediction of severe dengue has not been comprehensively reviewed before our study.

The 2011 WHO guidelines for dengue management highlight the presence of plasma leakage to differentiate between DF and DHF/DSS [50].

The 3 mm threshold for GBWT has been used in many studies since it was first described in the literature as a key indicator for plasma leakage and the prediction of severe dengue. Early ultrasonographic studies of dengue patients found the threshold. These studies showed a strong link between high GBWT and severe disease symptoms like low blood pressure and plasma leakage [15, 56].The consistency of this cut-off across various demographic and geographic contexts illustrates its utility in early risk stratification for therapeutic purposes. However, various organizations have suggested alternative criteria, like 5 mm, to improve diagnostic specificity [16]. These variations may arise due to differences in patient characteristics, ultrasound techniques, or comorbid conditions affecting GBWT. Additional research is required to validate these criteria across larger populations to enhance their predictive utility for severe dengue. Plasma leakage can be detected through GBWT at the first stages before it manifests as clinical symptoms and progresses to critical phase and shock [39]. Plasma leakage is a significant contributor to dengue-related mortality and remains the leading cause of severe complications [57, 58]. GBWT serves as an early marker of plasma leakage, often preceding the appearance of ascites, pleural effusion, and serological confirmation of IgM. Ultrasonography can detect GBWT as early as the 2nd or 3rd day of febrile illness [16, 38, 39]. Plasma leakage and GBWT are temporary events in the course of dengue disease [26, 38, 59]. GBWT resolves spontaneously at approximately the same time as the other markers of plasma leakage-ascites and pleural effusions [38]. However, ascites and pleural effusion might be absent before fluid therapy, even in severe shock syndrome [50]. These findings, alongside with relatively high sensitivity (88%) of GBWT, highlight the importance of GBWT as a predictor of dengue severity and an early warning sign of plasma leakage. The underlying mechanisms driving plasma leakage are not fully understood but are hypothesized to involve multiple pathways: (1) elevated cell-mediated and humoral immune response to dengue disease, especially in cases of dengue reinfection; (2) secretion of pro-inflammatory molecules (including tumor necrosis factor α, interleukin 6, interleukin 8 and platelet activating factor); (3) destruction of the endothelial glycocalyx due to dengue virus non-structural protein-1 (NS1); and (4) formation of excessive reactive oxygen species [59–61]. The results of our study support this because they show that GBWT is an early sign of plasma leakage that is linked to basic processes like immune system reactions and endothelial damage caused by DF.

Tsheten et al. [54] and Yuan et al. [8] have previously shown that ultrasound markers such as pleural effusion (OR = 5.72–15.84), ascites (OR = 6.30–24.30), and hepatomegaly (OR = 4.40–5.92) have strong association with severe dengue. In comparison, we estimated that GBWT has odds ratio of 2.65 for prediction of severe dengue. Strong association of these markers with the severity of dengue highlights the clinical utility of ultrasound imaging in follow-up of dengue patients.

However, detection and follow-up of severe dengue patients using GBWT alone may result in overdiagnosis, since GBWT is a non-specific finding and can result in false-positive estimations [11]. GBWT can be caused by several conditions other than dengue disease, such as cholecystitis, liver diseases, heart failure [62], leptospirosis [63], and malaria infection [64]. In our meta-analysis, even if some of the included studies had excluded patients with comorbidities that could have potentially resulted in GBWT, the specificity of GBWT for the prediction of severe dengue was estimated at 63%, which is relatively low. However, making some adjustments in the assessment of GBWT might increase its specificity for detection and follow-up of severe dengue disease, including fasting for 4–6 h prior to ultrasonography [11, 41, 42], raising the thickening threshold from 3 to 5 mm [13], and recognizing certain patterns of GBWT such as the “honeycomb” pattern [16, 41]. In confirmed cases of dengue disease, GBWT can serve as a predictive factor for the severity of dengue considering its high sensitivity for the prediction of severe dengue; however, it should be employed with increased caution, especially in patients with comorbidities affecting GBWT, and in patients without definitive dengue diagnosis.

We suggest that GBWT might be a valuable tool for monitoring the progression of severe dengue [27, 38]. The detection of GBWT is cost-effective, safe, sensitive, and readily available through ultrasonography at the patients’ bedside [36, 38, 50]. Serial evaluation of GBWT could be integrated as a standard part of treatment protocols for hospitalized patients with dengue [38, 42]. Even in mild cases, the presence of GBWT should raise suspicion for progression to severe dengue [16, 38]. We also suggest that GBWT might be associated with the prognosis of dengue disease, and its incorporation into dengue management may reduce the risk of overlooking potentially severe cases and dengue-related mortality [36]. However, further studies are necessary to characterize the association between GBWT progression and the outcome of dengue disease.

There are certain limitations to our study. Most of the participants included in the study were hospitalized patients, which may not represent outpatient cases of dengue disease. Therefore, the general population of dengue patients may not benefit from our findings. Some of the included studies in our review excluded patients with comorbidities that could potentially affect GBWT, while others did not. We are uncertain about the overall effect of this on our overall analysis. The included studies were substantially heterogeneous in terms of diagnostic and management criteria. Nevertheless, our subgroup analysis showed a uniform pattern of GBWT association with dengue severity, regardless of severity classification criteria. The heterogeneity of the study structures prevented us from tracking GBWT's onset and progression. Future research could address this issue.

## Conclusion

Severe dengue is associated with higher GBWT. The presence of GBWT in the context of dengue disease, even in mild cases, should raise suspicion for worsening in dengue severity. Using GBWT to detect cases with greater potential for severity may improve patient care and outcomes. However, we should approach the interpretation of GBWT carefully, taking into account its numerous potential differential diagnoses and low specificity.

## Supplementary Information

Supplementary Material 1: Supplementary Figures

Supplementary Material 2: Table S1

Supplementary Material 3: Table S2

Supplementary Material 4: Table S3

Supplementary Material 5: Table S4

## Data Availability

All data generated or analyzed during this study are included in this published article and its supplementary information files.
