# Supplementary material for: Ultrasound evaluation of gallbladder wall thickness for predicting severe dengue: a systematic review and meta-analysis
Source: Ultrasound J. 2025 Feb 3;17:12. doi: 10.1186/s13089-025-00417-5 (PMC11790530; doi:10.1186/s13089-025-00417-5)
Supplement: Supplementary file 1 — Supplementary Material 1: Supplementary Figures [file 13089_2025_417_MOESM1_ESM.docx]

**
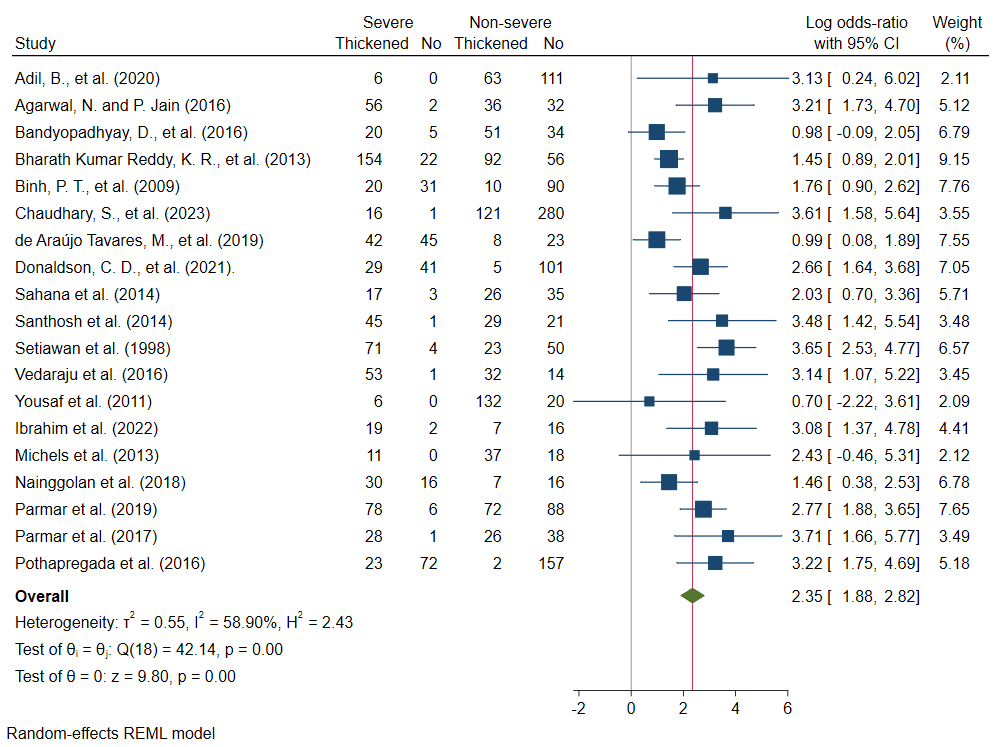
**

**Figure 2.** Overall analysis results. It indicates the correlation between gallbladder wall thickening (GBWT) and the severity of dengue. An odds ratio (OR) of 2.35 (95% CI: 1.88–2.82, p < 0.001) shows that people with severe dengue have a significantly higher GBWT. This means that there is a strong and statistically significant link between GBWT and disease severity.

**
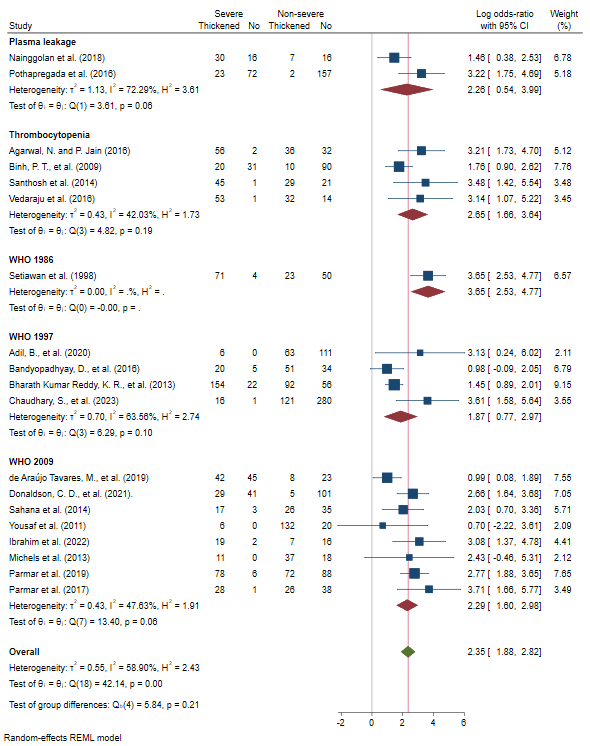
**

**Figure 3.** Subgroup analysis. It presents the results of subgroup analyses based on various severity criteria, including thrombocytopenia, plasma leakage, and WHO classifications (1986, 1997, and 2009). The findings align with the overall analysis, with ORs such as 2.65 (95% CI: 1.66–3.64) for thrombocytopenia and 2.26 (95% CI: 0.54–3.99) for plasma leakage, further supporting the correlation between GBWT and severe dengue.

**
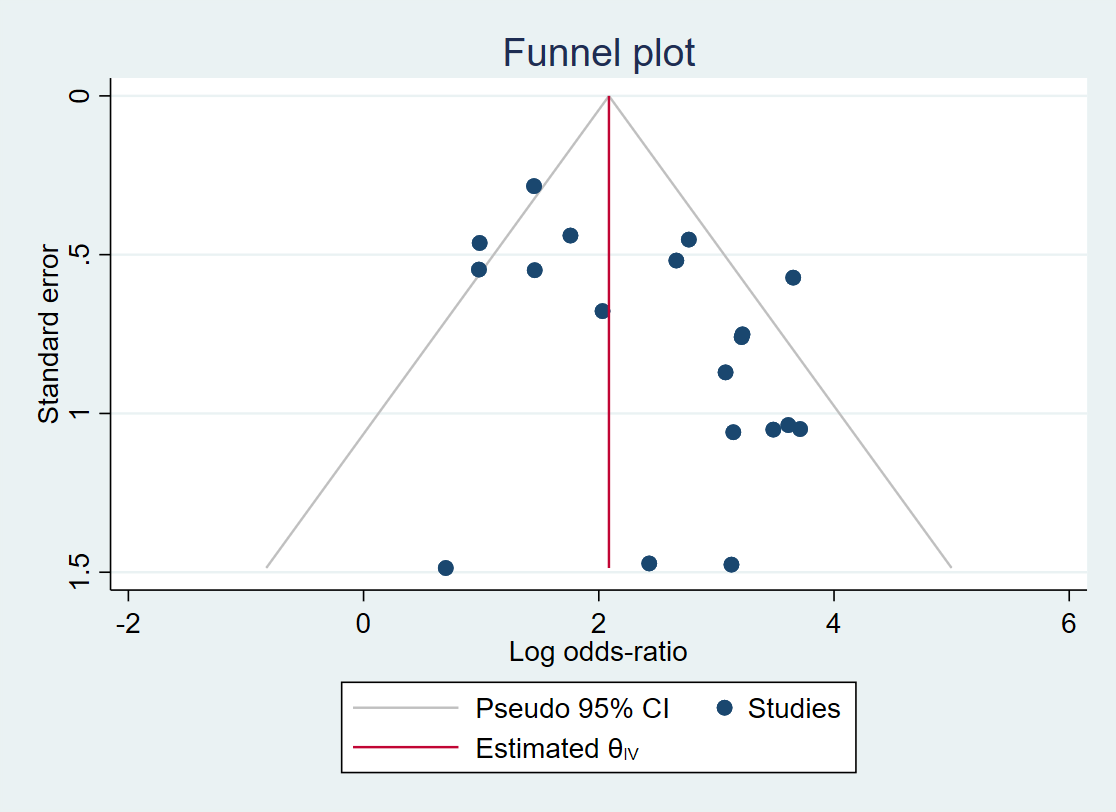
**

**Figure 4.** Funnel plot for publication bias assessment. The plot's symmetrical distribution of data points shows that there is no indication that publication bias impacts the meta-analysis's outcomes.

**
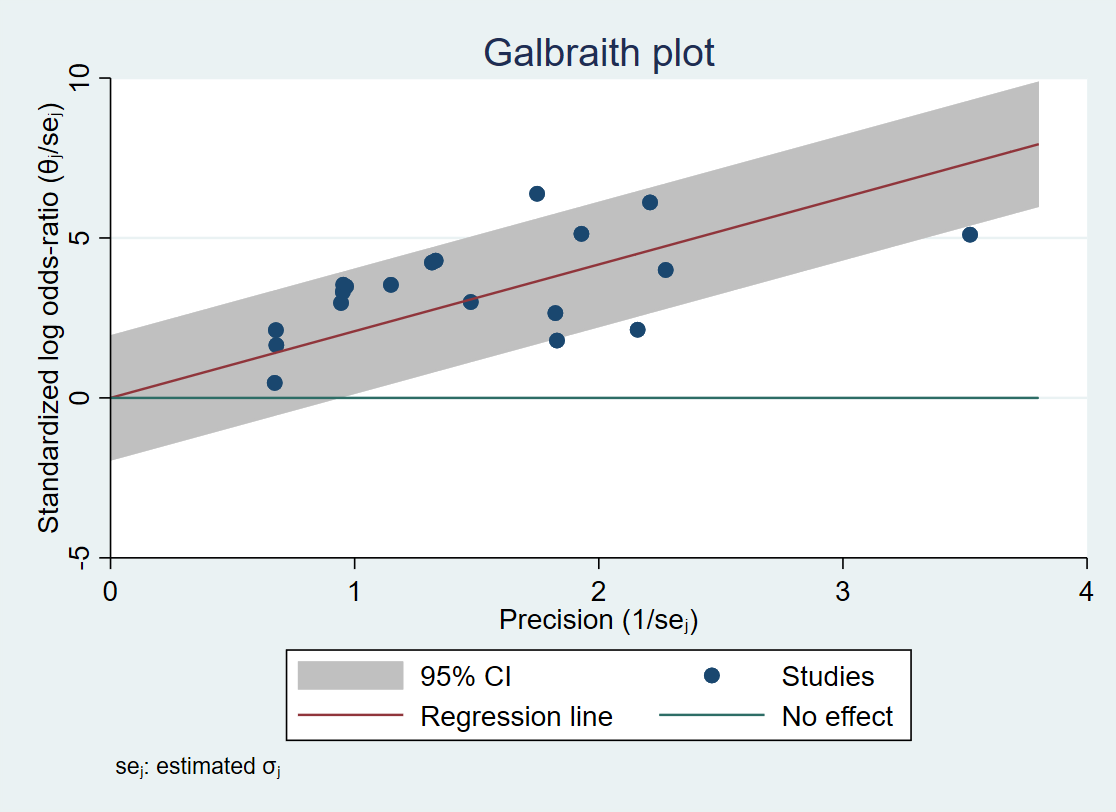
**

**Figure 5.** Galbraith plot for heterogeneity assessment. The plot identifies only a few minor outlier studies, and their impact on the overall results is minimal.


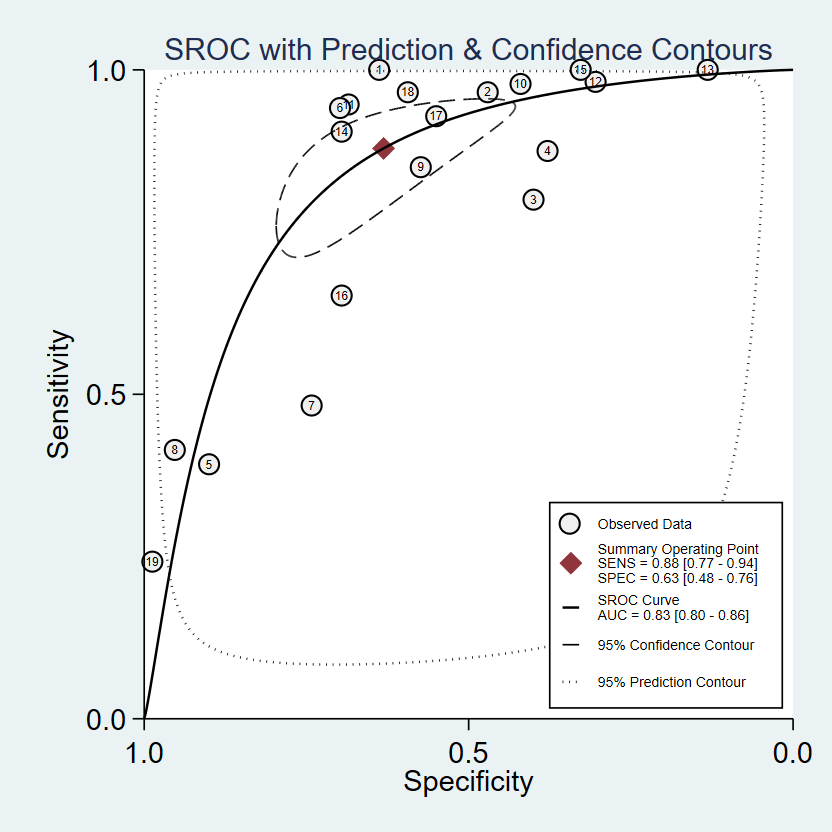
 **Figure 6**. Receiver Operating Characteristic (ROC) curve for the association of GBWT with severe dengue. It illustrates the discriminative ability of GBWT in predicting severe dengue, with an area under the curve (AUC) indicating high sensitivity and low specificity.


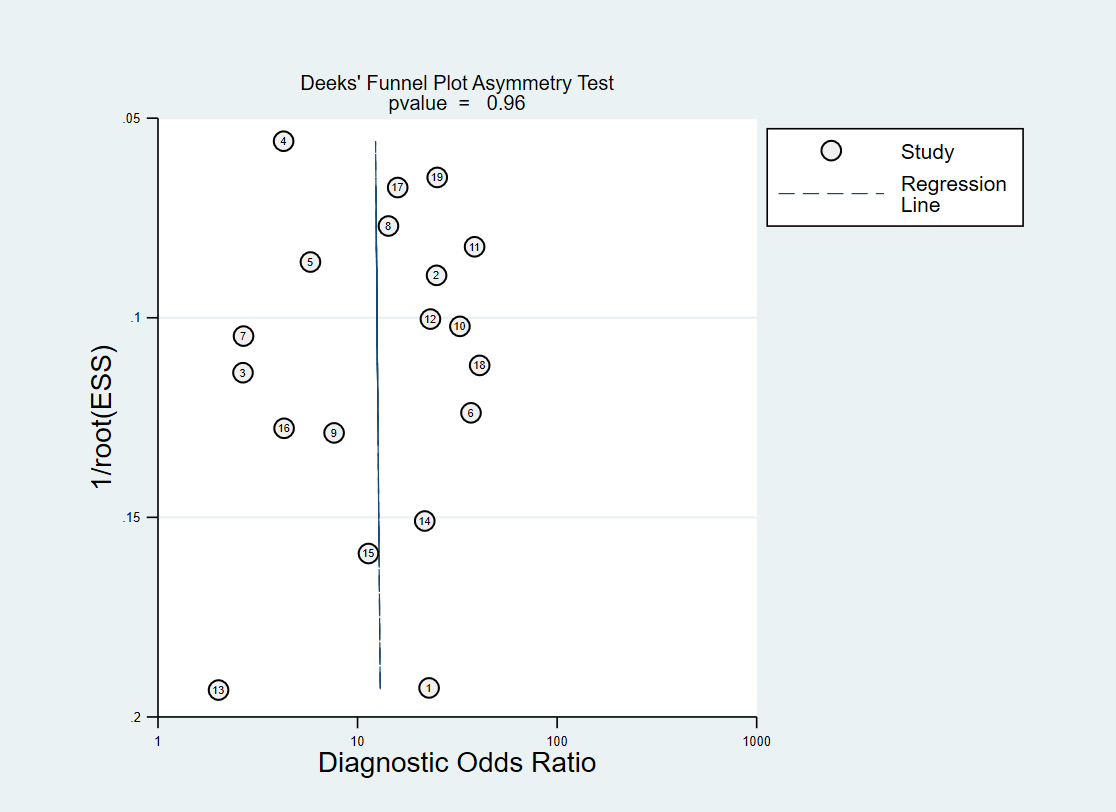
 **Figure 7.** Deeks' funnel plot for assessing publication bias in the association of GBWT with severe dengue. No significant asymmetry detected (p = 0.96), indicating minimal evidence of publication bias in the included studies.


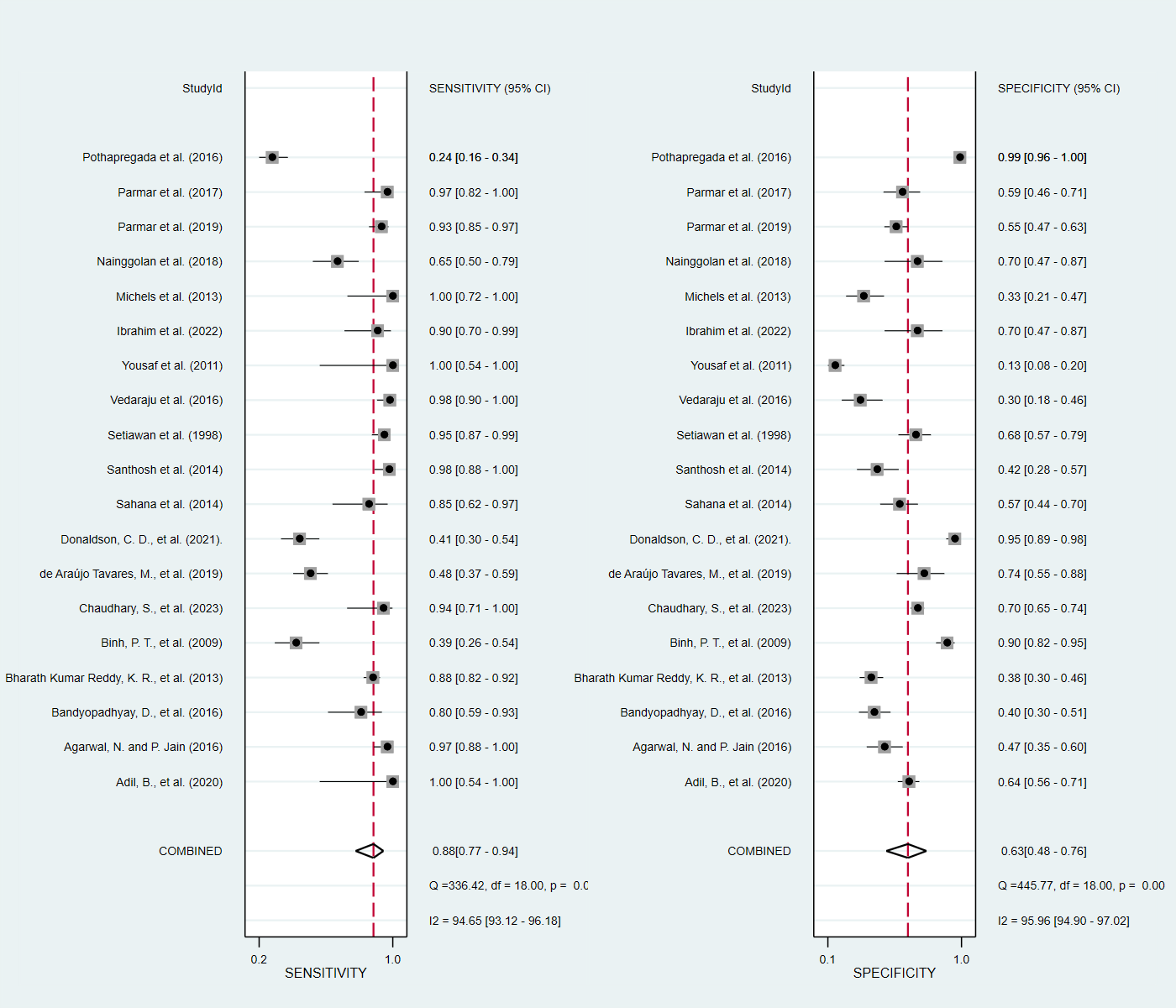
**Figure 8.** Forest plots for sensitivity and specificity of GBWT in predicting severe dengue. The pooled sensitivity of 0.88 (95% CI:0.77-0.94) and specificity of 0.63 (95% CI: 0.48-0.76) show that GBWT is present in most of severe dengue patients, however, it is non-specific.


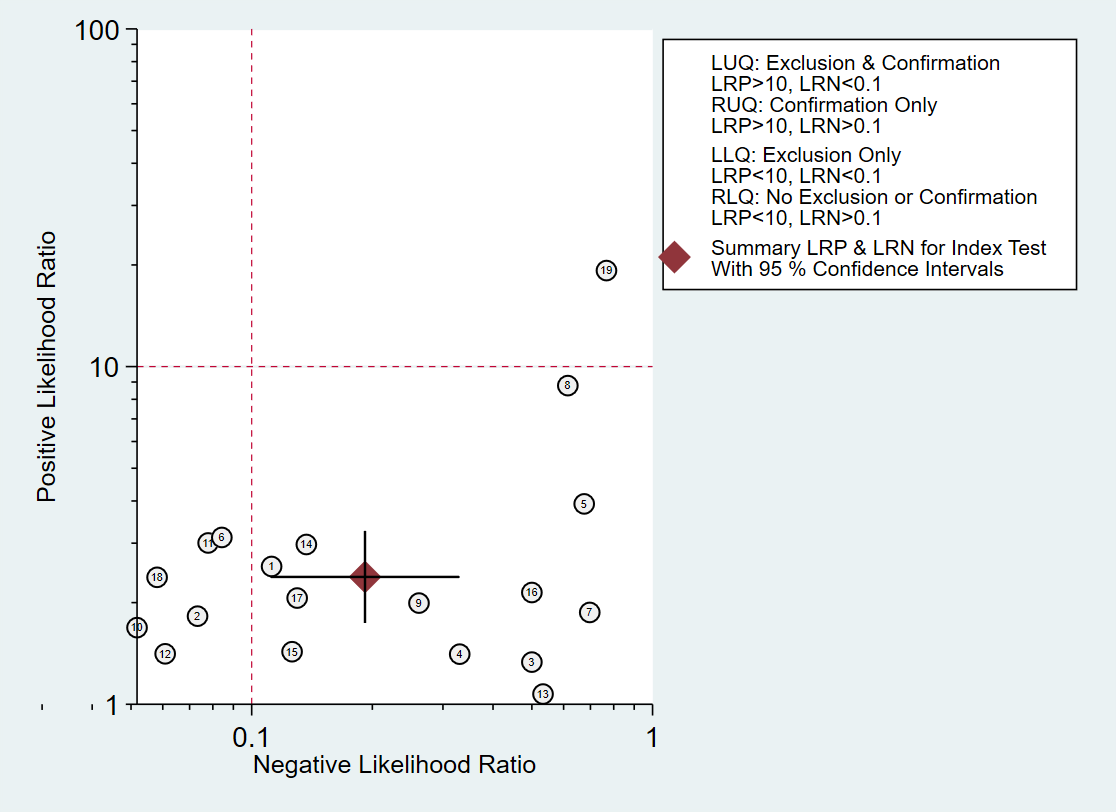


**Figure 9.** Likelihood ratio scatter plot for diagnostic accuracy. The plot demonstrates the pooled positive and negative likelihood ratios (with 95% confidence intervals), indicating that GBWT cannot be alone utilized to confirm or exclude severe dengue.


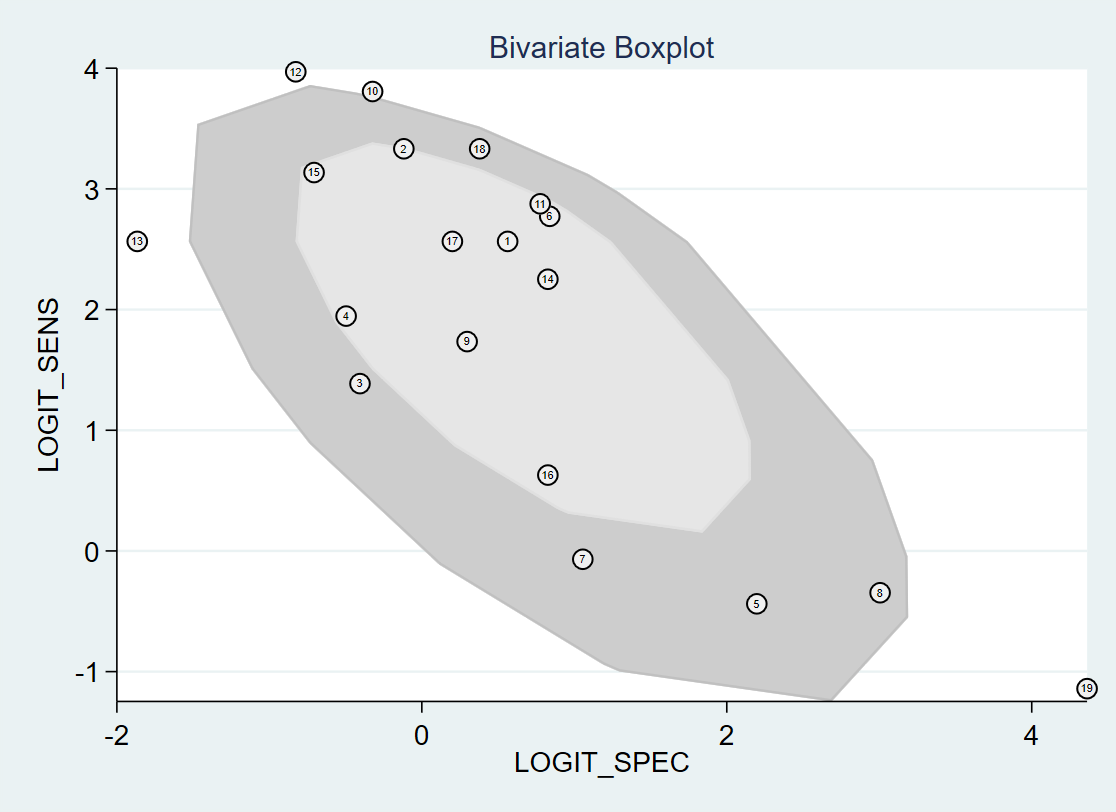


**Figure 10.** Bivariate boxplot for sensitivity and specificity. The plot illustrates the distribution of logit-transformed sensitivity and specificity across studies, with most points falling within the central area, indicating minimal outliers and consistent diagnostic performance.


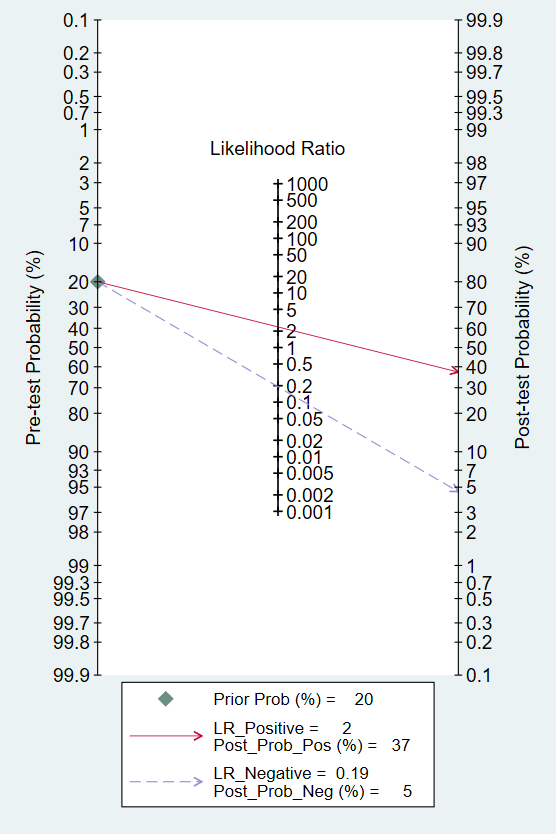


**Figure 11.** Fagan's nomogram for diagnostic test performance. It demonstrates how pre-test probability (20%) is updated to post-test probabilities of 37% and 5% based on positive and negative likelihood ratios.
