# Supplementary material for: Ultrasound evaluation of gallbladder wall thickness for predicting severe dengue: a systematic review and meta-analysis
Source: Ultrasound J. 2025 Feb 3;17:12. doi: 10.1186/s13089-025-00417-5 (PMC11790530; doi:10.1186/s13089-025-00417-5)
Supplement: Supplementary file 2 — Supplementary Material 2: Table S1 [file 13089_2025_417_MOESM2_ESM.docx]

**Supplementary file, Table S1**

**Table S1.** Search term for each database.

| 1. **PubMed:** 155 (2 August, 2024) |
| --- |
| (“Severe Dengue” OR "Dengue, Severe" OR "Dengues, Severe" OR "Severe Dengues" OR "Dengue Hemorrhagic Fever" OR "Hemorrhagic Dengue" OR "Hemorrhagic Dengues" OR "Hemorrhagic Fever, Dengue" OR "Singapore Hemorrhagic Fever" OR "Fever, Singapore Hemorrhagic" OR "Thai Hemorrhagic Fever" OR "Fever, Thai Hemorrhagic" OR "Philippine Hemorrhagic Fever" OR "Fever, Philippine Hemorrhagic" OR "Dengue Shock Syndrome" OR dengue OR "Dengue Fever" OR "Fever, Dengue" OR "Classical Dengue" OR "Classical Dengues" OR "Dengue, Classical" OR "Classical Dengue Fever" OR "Classical Dengue Fevers" OR "Dengue Fever, Classical" OR "Break-Bone Fever" OR "Break Bone Fever" OR "Fever, Break-Bone" OR "Breakbone Fever" OR "Fever, Breakbone" “dengue shock syndrome” OR “dengue complication*” OR “dengue death*” OR “dengue mortalit*” OR “dengue fatalit*” OR “dengue intensive care”) AND (("Acalculous Cholecystitis"[Mesh] OR “acalculous acute cholecystitis” [All Fields] OR “acalculous cholecystitis” [All Fields] OR “Acalculous Gallbladder Inflammation” [All Fields] OR "gallbladder"[MeSH Terms] OR "gallbladder"[All Fields] OR "gallbladders"[All Fields] OR "gallbladder s"[All Fields]) AND ("wall"[All Fields] OR "thicken"[All Fields] OR "thickened"[All Fields] OR "thickening"[All Fields] OR "thickenings"[All Fields] OR "thickens"[All Fields]) OR ("Ultrasonography" OR "Echotomography" OR "Ultrasonic Imaging" OR "Imaging, Ultrasonic" OR "Sonography, Medical" OR "Medical Sonography" OR "Ultrasound Imaging" OR "Imagings, Ultrasound" OR "Imaging, Ultrasound" OR "Ultrasonographic Imaging" OR "Imagings, Ultrasonographic" OR "Imaging, Ultrasonographic" OR "Ultrasonographic Imagings" OR "Echography" OR "Diagnostic Ultrasound" OR "Diagnostic Ultrasounds" OR "Ultrasound, Diagnostic" OR "Ultrasounds, Diagnostic" OR "Echotomography, Computer" OR "Computer Echotomography" OR "Tomography, Ultrasonic" OR "Ultrasonic Tomography" OR "Diagnosis, Ultrasonic" OR "Diagnoses, Ultrasonic" OR "Ultrasonic Diagnoses" OR "Ultrasonic Diagnosis" OR "ultrasound") OR ("Tomography, X-Ray Computed" OR "X-Ray Computed Tomography" OR "Tomodensitometry" OR "Tomography, X Ray Computed" OR "X Ray Tomography, Computed" OR "X-Ray Tomography, Computed" OR "Computed X-Ray Tomography" OR "Tomographies, Computed X-Ray" OR "Tomography, Computed X-Ray" OR "Tomography, Xray Computed" OR "Computed Tomography, Xray" OR "Xray Computed Tomography" OR "CAT Scan, X Ray" OR "CAT Scan, X-Ray" OR "CAT Scans, X-Ray" OR "Scans, X-Ray CAT" OR "Scan, X-Ray CAT" OR "X-Ray CAT Scan" OR "X-Ray CAT Scans" OR "Tomography, Transmission Computed" OR "Computed Tomography, Transmission" OR "Transmission Computed Tomography" OR "CT Scan, X-Ray" OR "CT Scans, X-Ray" OR "CT Scan, X Ray" OR "Scans, X-Ray CT" OR "Scan, X-Ray CT" OR "X-Ray CT Scan" OR "X-Ray CT Scans" OR "Tomography, X-Ray Computerized" OR "Tomography, X Ray Computerized" OR "Computerized Tomography, X Ray" OR "Computerized Tomography, X-Ray" OR "X-Ray Computerized Tomography" OR "Tomography, X-Ray Computer Assisted" OR "Tomography, X Ray Computer Assisted" OR "Computed Tomography, X-Ray" OR "Computed Tomography, X Ray" OR "X Ray Computerized Tomography" OR "Computed X Ray Tomography" OR "CT X Ray" OR "CT X Rays" OR "X Ray, CT" OR "X Rays, CT" OR "X-Ray Computer Assisted Tomography" OR "X Ray Computer Assisted Tomography" OR "Cine-CT" OR "Cine CT" OR "Electron Beam Computed Tomography" OR "Electron Beam Tomography" OR "Beam Tomography, Electron" OR "Tomography, Electron Beam" OR "Tomography, X-Ray Computerized Axial" OR "Tomography, X Ray Computerized Axial" OR "X-Ray Computerized Axial Tomography" OR "X Ray Computerized Axial Tomography" OR "CT Scan" OR "CT Scans" OR "Computed Tomography" OR "CT Imaging" OR "CT Imaging, Diagnostic" OR "Diagnostic CT Scan" OR "Diagnostic CT Scans")) |
| 1. **Embase:** 42 (2 August, 2024) |
| (('severe dengue':ab,ti OR 'dengue, severe':ab,ti OR 'dengues, severe':ab,ti OR 'severe dengues':ab,ti OR 'dengue hemorrhagic fever':ab,ti OR 'hemorrhagic dengue':ab,ti OR 'hemorrhagic dengues':ab,ti OR 'hemorrhagic fever, dengue':ab,ti OR 'singapore hemorrhagic fever':ab,ti OR 'fever, singapore hemorrhagic':ab,ti OR 'thai hemorrhagic fever':ab,ti OR 'fever, thai hemorrhagic':ab,ti OR 'philippine hemorrhagic fever':ab,ti OR 'fever, philippine hemorrhagic':ab,ti OR 'dengue shock syndrome':ab,ti OR dengue:ab,ti OR 'dengue fever':ab,ti OR 'fever, dengue':ab,ti OR 'classical dengue':ab,ti OR 'classical dengues':ab,ti OR 'dengue, classical':ab,ti OR 'classical dengue fever':ab,ti OR 'classical dengue fevers':ab,ti OR 'dengue fever, classical':ab,ti OR 'break-bone fever':ab,ti OR 'break bone fever':ab,ti OR 'fever, break-bone':ab,ti OR 'breakbone fever':ab,ti OR 'fever, breakbone':ab,ti) AND ('dengue shock syndrome':ab,ti OR 'dengue complication*':ab,ti OR 'dengue death*':ab,ti OR 'dengue mortalit*':ab,ti OR 'dengue fatalit*':ab,ti OR 'dengue intensive care':ab,ti) AND (('Acalculous Cholecystitis'/exp OR 'acalculous acute cholecystitis' OR 'acalculous cholecystitis' OR 'Acalculous Gallbladder Inflammation' OR 'gallbladder':ab,ti OR 'gallbladders':ab,ti OR 'gallbladder s':ab,ti) AND ('wall':ab,ti OR 'thicken':ab,ti OR 'thickened':ab,ti OR 'thickening':ab,ti OR 'thickenings':ab,ti OR 'thickens':ab,ti) OR 'ultrasonography':ab,ti OR 'echotomography':ab,ti OR 'ultrasonic imaging':ab,ti OR 'imaging, ultrasonic':ab,ti OR 'sonography, medical':ab,ti OR 'medical sonography':ab,ti OR 'ultrasound imaging':ab,ti OR 'imagings, ultrasound':ab,ti OR 'imaging, ultrasound':ab,ti OR 'ultrasonographic imaging':ab,ti OR 'imagings, ultrasonographic':ab,ti OR 'imaging, ultrasonographic':ab,ti OR 'ultrasonographic imagings':ab,ti OR 'echography':ab,ti OR 'diagnostic ultrasound':ab,ti OR 'diagnostic ultrasounds':ab,ti OR 'ultrasound, diagnostic':ab,ti OR 'ultrasounds, diagnostic':ab,ti OR 'echotomography, computer':ab,ti OR 'computer echotomography':ab,ti OR 'tomography, ultrasonic':ab,ti OR 'ultrasonic tomography':ab,ti OR 'diagnosis, ultrasonic':ab,ti OR 'diagnoses, ultrasonic':ab,ti OR 'ultrasonic diagnoses':ab,ti OR 'ultrasonic diagnosis':ab,ti OR 'ultrasound':ab,ti OR 'tomography, x-ray computed':ab,ti OR 'x-ray computed tomography':ab,ti OR 'tomodensitometry':ab,ti OR 'tomography, x ray computed':ab,ti OR 'x ray tomography, computed':ab,ti OR 'x-ray tomography, computed':ab,ti OR 'computed x-ray tomography':ab,ti OR 'tomographies, computed x-ray':ab,ti OR 'tomography, computed x-ray':ab,ti OR 'tomography, xray computed':ab,ti OR 'computed tomography, xray':ab,ti OR 'xray computed tomography':ab,ti OR 'cat scan, x ray':ab,ti OR 'cat scan, x-ray':ab,ti OR 'cat scans, x-ray':ab,ti OR 'scans, x-ray cat':ab,ti OR 'scan, x-ray cat':ab,ti OR 'x-ray cat scan':ab,ti OR 'x-ray cat scans':ab,ti OR 'tomography, transmission computed':ab,ti OR 'computed tomography, transmission':ab,ti OR 'transmission computed tomography':ab,ti OR 'ct scan, x-ray':ab,ti OR 'ct scans, x-ray':ab,ti OR 'ct scan, x ray':ab,ti OR 'scans, x-ray ct':ab,ti OR 'scan, x-ray ct':ab,ti OR 'x-ray ct scan':ab,ti OR 'x-ray ct scans':ab,ti OR 'tomography, x-ray computerized':ab,ti OR 'tomography, x ray computerized':ab,ti OR 'x-ray computerized tomography':ab,ti OR 'x ray computerized tomography':ab,ti OR 'ct scan':ab,ti OR 'ct scans':ab,ti OR 'computed tomography':ab,ti OR 'ct imaging':ab,ti OR 'ct imaging, diagnostic':ab,ti OR 'diagnostic ct scan':ab,ti OR 'diagnostic ct scans':ab,ti) |
| 1. **Scopus:** 96 (2 August, 2024) |
| ALL ( ( "Severe Dengue" OR "Dengue, Severe" OR "Dengues, Severe" OR "Severe Dengues" OR "Dengue Hemorrhagic Fever" OR "Hemorrhagic Dengue" OR "Hemorrhagic Dengues" OR "Hemorrhagic Fever, Dengue" OR "Singapore Hemorrhagic Fever" OR "Fever, Singapore Hemorrhagic" OR "Thai Hemorrhagic Fever" OR "Fever, Thai Hemorrhagic" OR "Philippine Hemorrhagic Fever" OR "Fever, Philippine Hemorrhagic" OR "Dengue Shock Syndrome" OR dengue OR "Dengue Fever" OR "Fever, Dengue" OR "Classical Dengue" OR "Classical Dengues" OR "Dengue, Classical" OR "Classical Dengue Fever" OR "Classical Dengue Fevers" OR "Dengue Fever, Classical" OR "Break-Bone Fever" OR "Break Bone Fever" OR "Fever, Break-Bone" OR "Breakbone Fever" OR "Fever, Breakbone" "dengue shock syndrome" OR "dengue complication*" OR "dengue death*" OR "dengue mortalit*" OR "dengue fatalit*" OR "dengue intensive care" ) AND ( ("Acalculous Cholecystitis" OR "acalculous acute cholecystitis" OR "acalculous cholecystitis" OR "Acalculous Gallbladder Inflammation" OR gallbladder OR gallbladder OR gallbladders OR gallbladder AND s ) AND ( "wall" OR "thicken" OR "thickened" OR "thickening" OR "thickenings" OR "thickens" OR thick* ) OR ( "Ultrasonography" OR "Echotomography" OR "Ultrasonic Imaging" OR "Imaging, Ultrasonic" OR "Sonography, Medical" OR "Medical Sonography" OR "Ultrasound Imaging" OR "Imagings, Ultrasound" OR "Imaging, Ultrasound" OR "Ultrasonographic Imaging" OR "Imagings, Ultrasonographic" OR "Imaging, Ultrasonographic" OR "Ultrasonographic Imagings" OR "Echography" OR "Diagnostic Ultrasound" OR "Diagnostic Ultrasounds" OR "Ultrasound, Diagnostic" OR "Ultrasounds, Diagnostic" OR "Echotomography, Computer" OR "Computer Echotomography" OR "Tomography, Ultrasonic" OR "Ultrasonic Tomography" OR "Diagnosis, Ultrasonic" OR "Diagnoses, Ultrasonic" OR "Ultrasonic Diagnoses" OR "Ultrasonic Diagnosis" OR "ultrasound" ) OR ( "Tomography, X-Ray Computed" OR "X-Ray Computed Tomography" OR "Tomodensitometry" OR "Tomography, X Ray Computed" OR "X Ray Tomography, Computed" OR "X-Ray Tomography, Computed" OR "Computed X-Ray Tomography" OR "Tomographies, Computed X-Ray" OR "Tomography, Computed X-Ray" OR "Tomography, Xray Computed" OR "Computed Tomography, Xray" OR "Xray Computed Tomography" OR "CAT Scan, X Ray" OR "CAT Scan, X-Ray" OR "CAT Scans, X-Ray" OR "Scans, X-Ray CAT" OR "Scan, X-Ray CAT" OR "X-Ray CAT Scan" OR "X-Ray CAT Scans" OR "Tomography, Transmission Computed" OR "Computed Tomography, Transmission" OR "Transmission Computed Tomography" OR "CT Scan, X-Ray" OR "CT Scans, X-Ray" OR "CT Scan, X Ray" OR "Scans, X-Ray CT" OR "Scan, X-Ray CT" OR "X-Ray CT Scan" OR "X-Ray CT Scans" OR "Tomography, X-Ray Computerized" OR "Tomography, X Ray Computerized" OR "Computerized Tomography, X Ray" OR "Computerized Tomography, X-Ray" OR "X-Ray Computerized Tomography" OR "Tomography, X-Ray Computer Assisted" OR "Tomography, X Ray Computer Assisted" OR "Computed Tomography, X-Ray" OR "Computed Tomography, X Ray" OR "X Ray Computerized Tomography" OR "Computed X Ray Tomography" OR "CT X Ray" OR "CT X Rays" OR "X Ray, CT" OR "X Rays, CT" OR "X-Ray Computer Assisted Tomography" OR "X Ray Computer Assisted Tomography" OR "Cine-CT" OR "Cine CT" OR "Electron Beam Computed Tomography" OR "Electron Beam Tomography" OR "Beam Tomography, Electron" OR "Tomography, Electron Beam" OR "Tomography, X-Ray Computerized Axial" OR "Tomography, X Ray Computerized Axial" OR "X-Ray Computerized Axial Tomography" OR "X Ray Computerized Axial Tomography" OR "CT Scan" OR "CT Scans" OR "Computed Tomography" OR "CT Imaging" OR "CT Imaging, Diagnostic" OR "Diagnostic CT Scan" OR "Diagnostic CT Scans" ) ) ) |
| 1. **Web of Science:** 242 (2 August, 2024) |
| TI = ((“Severe Dengue” OR "Dengue, Severe" OR "Dengues, Severe" OR "Severe Dengues" OR "Dengue Hemorrhagic Fever" OR "Hemorrhagic Dengue" OR "Hemorrhagic Dengues" OR "Hemorrhagic Fever, Dengue" OR "Singapore Hemorrhagic Fever" OR "Fever, Singapore Hemorrhagic" OR "Thai Hemorrhagic Fever" OR "Fever, Thai Hemorrhagic" OR "Philippine Hemorrhagic Fever" OR "Fever, Philippine Hemorrhagic" OR "Dengue Shock Syndrome" OR dengue OR "Dengue Fever" OR "Fever, Dengue" OR "Classical Dengue" OR "Classical Dengues" OR "Dengue, Classical" OR "Classical Dengue Fever" OR "Classical Dengue Fevers" OR "Dengue Fever, Classical" OR "Break-Bone Fever" OR "Break Bone Fever" OR "Fever, Break-Bone" OR "Breakbone Fever" OR "Fever, Breakbone" “dengue shock syndrome” OR “dengue complication*” OR “dengue death*” OR “dengue mortalit*” OR “dengue fatalit*” OR “dengue intensive care”) AND (("gallbladder"[MeSH Terms] OR "gallbladder"[All Fields] OR "gallbladders"[All Fields] OR "gallbladder s"[All Fields]) AND ("wall"[All Fields] OR "thicken"[All Fields] OR "thickened"[All Fields] OR "thickening"[All Fields] OR "thickenings"[All Fields] OR "thickens"[All Fields]) OR ("Ultrasonography" OR "Echotomography" OR "Ultrasonic Imaging" OR "Imaging, Ultrasonic" OR "Sonography, Medical" OR "Medical Sonography" OR "Ultrasound Imaging" OR "Imagings, Ultrasound" OR "Imaging, Ultrasound" OR "Ultrasonographic Imaging" OR "Imagings, Ultrasonographic" OR "Imaging, Ultrasonographic" OR "Ultrasonographic Imagings" OR "Echography" OR "Diagnostic Ultrasound" OR "Diagnostic Ultrasounds" OR "Ultrasound, Diagnostic" OR "Ultrasounds, Diagnostic" OR "Echotomography, Computer" OR "Computer Echotomography" OR "Tomography, Ultrasonic" OR "Ultrasonic Tomography" OR "Diagnosis, Ultrasonic" OR "Diagnoses, Ultrasonic" OR "Ultrasonic Diagnoses" OR "Ultrasonic Diagnosis" OR "ultrasound") OR ("Tomography, X-Ray Computed" OR "X-Ray Computed Tomography" OR "Tomodensitometry" OR "Tomography, X Ray Computed" OR "X Ray Tomography, Computed" OR "X-Ray Tomography, Computed" OR "Computed X-Ray Tomography" OR "Tomographies, Computed X-Ray" OR "Tomography, Computed X-Ray" OR "Tomography, Xray Computed" OR "Computed Tomography, Xray" OR "Xray Computed Tomography" OR "CAT Scan, X Ray" OR "CAT Scan, X-Ray" OR "CAT Scans, X-Ray" OR "Scans, X-Ray CAT" OR "Scan, X-Ray CAT" OR "X-Ray CAT Scan" OR "X-Ray CAT Scans" OR "Tomography, Transmission Computed" OR "Computed Tomography, Transmission" OR "Transmission Computed Tomography" OR "CT Scan, X-Ray" OR "CT Scans, X-Ray" OR "CT Scan, X Ray" OR "Scans, X-Ray CT" OR "Scan, X-Ray CT" OR "X-Ray CT Scan" OR "X-Ray CT Scans" OR "Tomography, X-Ray Computerized" OR "Tomography, X Ray Computerized" OR "Computerized Tomography, X Ray" OR "Computerized Tomography, X-Ray" OR "X-Ray Computerized Tomography" OR "Tomography, X-Ray Computer Assisted" OR "Tomography, X Ray Computer Assisted" OR "Computed Tomography, X-Ray" OR "Computed Tomography, X Ray" OR "X Ray Computerized Tomography" OR "Computed X Ray Tomography" OR "CT X Ray" OR "CT X Rays" OR "X Ray, CT" OR "X Rays, CT" OR "X-Ray Computer Assisted Tomography" OR "X Ray Computer Assisted Tomography" OR "Cine-CT" OR "Cine CT" OR "Electron Beam Computed Tomography" OR "Electron Beam Tomography" OR "Beam Tomography, Electron" OR "Tomography, Electron Beam" OR "Tomography, X-Ray Computerized Axial" OR "Tomography, X Ray Computerized Axial" OR "X-Ray Computerized Axial Tomography" OR "X Ray Computerized Axial Tomography" OR "CT Scan" OR "CT Scans" OR "Computed Tomography" OR "CT Imaging" OR "CT Imaging, Diagnostic" OR "Diagnostic CT Scan" OR "Diagnostic CT Scans")))  OR  AB = ((“Severe Dengue” OR "Dengue, Severe" OR "Dengues, Severe" OR "Severe Dengues" OR "Dengue Hemorrhagic Fever" OR "Hemorrhagic Dengue" OR "Hemorrhagic Dengues" OR "Hemorrhagic Fever, Dengue" OR "Singapore Hemorrhagic Fever" OR "Fever, Singapore Hemorrhagic" OR "Thai Hemorrhagic Fever" OR "Fever, Thai Hemorrhagic" OR "Philippine Hemorrhagic Fever" OR "Fever, Philippine Hemorrhagic" OR "Dengue Shock Syndrome" OR dengue OR "Dengue Fever" OR "Fever, Dengue" OR "Classical Dengue" OR "Classical Dengues" OR "Dengue, Classical" OR "Classical Dengue Fever" OR "Classical Dengue Fevers" OR "Dengue Fever, Classical" OR "Break-Bone Fever" OR "Break Bone Fever" OR "Fever, Break-Bone" OR "Breakbone Fever" OR "Fever, Breakbone" “dengue shock syndrome” OR “dengue complication*” OR “dengue death*” OR “dengue mortalit*” OR “dengue fatalit*” OR “dengue intensive care”) AND (("Acalculous Cholecystitis"[MeSH Terms] OR “acalculous acute cholecystitis” [All Fields] OR “acalculous cholecystitis” [All Fields] OR “Acalculous Gallbladder Inflammation” [All Fields] OR "gallbladder"[MeSH Terms] OR "gallbladder"[All Fields] OR "gallbladders"[All Fields] OR "gallbladder s"[All Fields]) AND ("wall"[All Fields] OR "thicken"[All Fields] OR "thickened"[All Fields] OR "thickening"[All Fields] OR "thickenings"[All Fields] OR "thickens"[All Fields]) OR ("Ultrasonography" OR "Echotomography" OR "Ultrasonic Imaging" OR "Imaging, Ultrasonic" OR "Sonography, Medical" OR "Medical Sonography" OR "Ultrasound Imaging" OR "Imagings, Ultrasound" OR "Imaging, Ultrasound" OR "Ultrasonographic Imaging" OR "Imagings, Ultrasonographic" OR "Imaging, Ultrasonographic" OR "Ultrasonographic Imagings" OR "Echography" OR "Diagnostic Ultrasound" OR "Diagnostic Ultrasounds" OR "Ultrasound, Diagnostic" OR "Ultrasounds, Diagnostic" OR "Echotomography, Computer" OR "Computer Echotomography" OR "Tomography, Ultrasonic" OR "Ultrasonic Tomography" OR "Diagnosis, Ultrasonic" OR "Diagnoses, Ultrasonic" OR "Ultrasonic Diagnoses" OR "Ultrasonic Diagnosis" OR "ultrasound") OR ("Tomography, X-Ray Computed" OR "X-Ray Computed Tomography" OR "Tomodensitometry" OR "Tomography, X Ray Computed" OR "X Ray Tomography, Computed" OR "X-Ray Tomography, Computed" OR "Computed X-Ray Tomography" OR "Tomographies, Computed X-Ray" OR "Tomography, Computed X-Ray" OR "Tomography, Xray Computed" OR "Computed Tomography, Xray" OR "Xray Computed Tomography" OR "CAT Scan, X Ray" OR "CAT Scan, X-Ray" OR "CAT Scans, X-Ray" OR "Scans, X-Ray CAT" OR "Scan, X-Ray CAT" OR "X-Ray CAT Scan" OR "X-Ray CAT Scans" OR "Tomography, Transmission Computed" OR "Computed Tomography, Transmission" OR "Transmission Computed Tomography" OR "CT Scan, X-Ray" OR "CT Scans, X-Ray" OR "CT Scan, X Ray" OR "Scans, X-Ray CT" OR "Scan, X-Ray CT" OR "X-Ray CT Scan" OR "X-Ray CT Scans" OR "Tomography, X-Ray Computerized" OR "Tomography, X Ray Computerized" OR "Computerized Tomography, X Ray" OR "Computerized Tomography, X-Ray" OR "X-Ray Computerized Tomography" OR "Tomography, X-Ray Computer Assisted" OR "Tomography, X Ray Computer Assisted" OR "Computed Tomography, X-Ray" OR "Computed Tomography, X Ray" OR "X Ray Computerized Tomography" OR "Computed X Ray Tomography" OR "CT X Ray" OR "CT X Rays" OR "X Ray, CT" OR "X Rays, CT" OR "X-Ray Computer Assisted Tomography" OR "X Ray Computer Assisted Tomography" OR "Cine-CT" OR "Cine CT" OR "Electron Beam Computed Tomography" OR "Electron Beam Tomography" OR "Beam Tomography, Electron" OR "Tomography, Electron Beam" OR "Tomography, X-Ray Computerized Axial" OR "Tomography, X Ray Computerized Axial" OR "X-Ray Computerized Axial Tomography" OR "X Ray Computerized Axial Tomography" OR "CT Scan" OR "CT Scans" OR "Computed Tomography" OR "CT Imaging" OR "CT Imaging, Diagnostic" OR "Diagnostic CT Scan" OR "Diagnostic CT Scans"))) |
