# Supplementary material for: Ultrasound evaluation of gallbladder wall thickness for predicting severe dengue: a systematic review and meta-analysis
Source: Ultrasound J. 2025 Feb 3;17:12. doi: 10.1186/s13089-025-00417-5 (PMC11790530; doi:10.1186/s13089-025-00417-5)
Supplement: Supplementary file 4 — Supplementary Material 4: Table S3 [file 13089_2025_417_MOESM4_ESM.docx]

| **Selection** | | | | | | **Comparability** | | | **Outcome** | | | |
| --- | --- | --- | --- | --- | --- | --- | --- | --- | --- | --- | --- | --- |
| **Study**  **First author (year)** | **Representativeness of the Exposed Cohort** | **Selection of the Non-Exposed Cohort** | **Ascertainment of Exposure** | **Demonstration That Outcome of Interest Was Not Present at Start of Study** | **Subtotal** | **Age** | **Other** | **Subtotal** | **Assessment of Outcome** | **Was Follow-Up Long Enough for Outcomes to Occur** | **Adequacy of Follow Up of Cohorts** | **Subtotal** |
| Sahana et al. [1]  (2014) | * | * | * | * | 4 | _ | _ | 0 | _ | * | * | 2 |
| Setiawan et al. [2]  (1995) | * | * | * | * | 4 | _ | _ | 0 | _ | * | * | 2 |
| Setiawan et al. [3]  (1998) | * | * | * | * | 4 | _ | _ | 0 | _ | * | * | 2 |
| Uthraraj et al. [4]  (2022) | * | * | * | * | 4 | _ | _ | 0 | _ | * | * | 2 |
| Adil, B., et al. [5]  (2020) | * | * | * | * | 4 | * | * | 2 | * | * | * | 3 |
| Agarwal, N. and P. Jain [6]  (2016) | * | * | * | * | 4 | _ | _ | 0 | _ | * | * | 2 |
| Bharath Kumar Reddy, K. R., et al. [7]  (2013) | * | * | * | * | 4 | _ | _ | 0 | * | * | * | 3 |
| Binh, P. T., et al. [8] (2009) | * | * | * | * | 3 | _ | _ | 0 | _ | * | * | 2 |
| Chacko, B. and G. Subramanian[9]  (2008) | * | * | _ | * | 3 | _ | _ | 0 | _ | * | _ | 1 |
| Chaudhary, S., et al. [10] (2023) | * | * | * | * | 4 | * | * | 2 | _ | * | * | 2 |
| Donaldson, C. D., et al. [11]  (2021) | * | * | * | * | 4 | _ | _ | 0 | _ | * | * | 2 |
| Mallhi et al. [12]  (2015) | * | * | * | * | 4 | _ | _ | 0 | _ | * | * | 2 |
| Michels et al. [13] (2013) | * | * | * | * | 4 | _ | _ | 0 | _ | * | * | 2 |
| Oliveira et al. [14] (2009) | * | * | * | * | 4 | _ | _ | 0 | _ | * | * | 2 |
| Osorio et al. [15]  (2023) | * | * | * | * | 4 | * | _ | 1 | * | * | * | 3 |
| Pothapregada et al. [16] (2016) | * | * | * | * | 4 | _ | _ | 0 | _ | * | * | 2 |

**References:**

1. Sahana, K.S. and R. Sujatha, *Clinical Profile of Dengue Among Children According to Revised WHO Classification: Analysis of a 2012 Outbreak from Southern India.* Indian Journal of Pediatrics, 2015. **82**(2): p. 109-113.

2. Setiawan, M.W., et al., *Gallbladder wall thickening in dengue hemorrhagic fever: an ultrasonographic study.* J Clin Ultrasound, 1995. **23**(6): p. 357-62.

3. Setiawan, M.W., et al., *Dengue haemorrhagic fever: Ultrasound as an aid to predict the severity of the disease.* PEDIATRIC RADIOLOGY, 1998. **28**(1): p. 1-4.

4. Uthraraj, N.S., et al., *Predictive Factors for the Complications of Dengue Fever in Children: A Retrospective Analysis.* Cureus, 2022. **14**(12): p. e33027.

5. Adil, B., et al., *Gall Bladder Wall Thickening in Dengue Fever - Aid in Labelling Dengue Hemorrhagic Fever and a Marker of Severity.* Cureus, 2020. **12**(11): p. e11331.

6. Agarwal, N. and P. Jain, *Sonography in Dengue Fever: An Adjunct to Clinico-laboratory Profile.* Indian Journal of Public Health Research & Development, 2016. **7**: p. 299.

7. Bharath Kumar Reddy, K.R., et al., *Ultrasonography as a tool in predicting the severity of dengue fever in children--a useful aid in a developing country.* Pediatr Radiol, 2013. **43**(8): p. 971-7.

8. Binh, P.T., et al., *Early clinical and biological features of severe clinical manifestations of dengue in Vietnamese adults.* Journal of Clinical Virology, 2009. **45**(4): p. 276-280.

9. Chacko, B. and G. Subramanian, *Clinical, laboratory and radiological parameters in children with dengue fever and predictive factors for dengue shock syndrome.* J Trop Pediatr, 2008. **54**(2): p. 137-40.

10. Chaudhary, S., et al., *Abdominal and Chest Ultrasonography: A predictor for disease progression in nonsevere dengue.* Medical Journal Armed Forces India, 2023. **79**(4): p. 386-391.

11. Donaldson, C.D., et al., *Admission ultrasonography as a predictive tool for thrombocytopenia and disease severity in dengue infection.* Trans R Soc Trop Med Hyg, 2021. **115**(12): p. 1396-1402.

12. Mallhi, T.H., et al., *Clinico-laboratory spectrum of dengue viral infection and risk factors associated with dengue hemorrhagic fever: A retrospective study.* BMC Infectious Diseases, 2015. **15**(1).

13. Michels, M., et al., *The Predictive Diagnostic Value of Serial Daily Bedside Ultrasonography for Severe Dengue in Indonesian Adults.* PLOS NEGLECTED TROPICAL DISEASES, 2013. **7**(6).

14. Oliveira, G.A., et al., *Transient reticular gallbladder wall thickening in severe dengue fever: a reliable sign of plasma leakage.* Pediatr Radiol, 2010. **40**(5): p. 720-4.

15. Osorio, L., et al., *Evaluation of remote radiologist-interpreted point-of-care ultrasound for suspected dengue patients in a primary health care facility in Colombia.* Infectious Diseases of Poverty, 2023. **12**(1).

16. Pothapregada, S., et al., *Is Ultrasound a Useful Tool to Predict Severe Dengue Infection?* Indian J Pediatr, 2016. **83**(6): p. 500-4.
