# Supplementary material for: Ultrasound evaluation of gallbladder wall thickness for predicting severe dengue: a systematic review and meta-analysis
Source: Ultrasound J. 2025 Feb 3;17:12. doi: 10.1186/s13089-025-00417-5 (PMC11790530; doi:10.1186/s13089-025-00417-5)
Supplement: Supplementary file 5 — Supplementary Material 5: Table S4 [file 13089_2025_417_MOESM5_ESM.docx]

| **Selection** | | | | | | **Comparability** | | | **Outcome** | | |  |
| --- | --- | --- | --- | --- | --- | --- | --- | --- | --- | --- | --- | --- |
| **Study**  **First author (year)** | **Representativeness of the Sample** | **Sample size** | **None-respondents** | **Ascertainment of the exposure** | **Subtotal** | **Age** | **Sex** | **Subtotal** | **Assessment of Outcome** | **Statistical test** | **Subtotal** |  |
| Asghar, M. S., et al. [1]  (2022) | * | * | * | ** | 5 | * | * | 2 | * | * | 2 | |
| Bandyopadhyay, D., et al. [2] (2016) | * | * | * | ** | 5 | _ | * | 1 | * | * | 2 | |
| de Araújo Tavares, M., et al. [3] (2019) | * | * | * | ** | 5 | * | * | 2 | * | * | 2 | |
| Venkata Sai et al. [4]  (2005) | * | * | * | ** | 5 | _ | _ | 0 | * | _ | 1 | |
| Santhosh et al. [5]  (2014) | * | * | * | ** | 5 | _ | _ | 0 | * | * | 2 | |
| Tauseef et al. [6]  (2019) | * | * | _ | ** | 4 | _ | _ | 0 | * | * | 2 | |
| Vedaraju et al. [7]  (2016) | * | * | * | ** | 5 | _ | * | 1 | * | * | 2 | |
| Yousaf et al. [8]  (2011) | * | * | * | ** | 5 | _ | * | 1 | * | * | 2 | |
| Zulkarnain et al. [9]  (2004) | * | * | * | ** | 5 | _ | _ | 0 | * | * | 2 | |
| Jain et al. [10]  (2024) | * | * | * | ** | 5 | _ | _ | 0 | * | * | 2 |  |
| Ibrahim et al. [11]  (2022) | * | * | * | ** | 5 | _ | _ | 0 | ** | * | 3 |  |
| Nainggolan et al. [12]  (2018) | * | * | * | ** | 5 | _ | * | 1 | ** | * | 3 |  |
| Parmar et al. [13]  (2017) | * | * | * | ** | 5 | _ | * | 1 | ** | * | 3 |  |
| Parmar et al. [14]  (2019) | * | * | * | ** | 5 | * | * | 2 | * | * | 2 |  |

**References:**

1. Asghar, M.S., et al., *Predictive Analysis of Gallbladder Wall Thickness as a Marker for Bleeding Risk and Need for Transfusion in Dengue Patients.* Jpn J Infect Dis, 2022. **75**(3): p. 234-240.

2. Bandyopadhyay, D., et al., *A study on spectrum of hepatobiliary dysfunctions and pattern of liver involvement in dengue infection.* Journal of Clinical and Diagnostic Research, 2016. **10**(5): p. OC21-OC26.

3. de Araújo Tavares, M., et al., *Clinical relevance of gallbladder wall thickening for dengue severity: A cross-sectional study.* PLoS ONE, 2019. **14**(8).

4. Sai, P.M.V., B. Dev, and R. Krishnan, *Role of ultrasound in dengue fever.* BRITISH JOURNAL OF RADIOLOGY, 2005. **78**(929): p. 416-418.

5. Santhosh, V.R., et al., *Sonography in the Diagnosis and Assessment of Dengue Fever.* JOURNAL OF CLINICAL IMAGING SCIENCE, 2014. **4**.

6. Tauseef, A., et al., *Role of Interleukin-10 and Abdominopelvic Ultrasound as a Potential Predictor of Disease Severity in Dengue Hemorrhagic Fever.* CUREUS JOURNAL OF MEDICAL SCIENCE, 2019. **11**(7).

7. Vedaraju, K.S., K.R.V. Kumar, and T.V. Vijayaraghavachari, *Role of Ultrasound in the Assessment of Dengue Fever.* INTERNATIONAL JOURNAL OF SCIENTIFIC STUDY, 2016. **3**(10): p. 59-62.

8. Yousaf, K.R., et al., *Sonographic Features of Polyserositis as an Adjunct to Clinico-Pathological Parameters in Diagnosing and Predicting the Severity of Dengue Fever.* PAKISTAN JOURNAL OF MEDICAL & HEALTH SCIENCES, 2011. **5**(1): p. 184-189.

9. Zulkarnain, I., *Gallbladder edema in Dengue hemorrhagic fever and its association with haematocrit levels and type of infections.* Acta Med Indones, 2004. **36**(2): p. 84-6.

10. Jain, A., et al., *A Cross-Sectional Study on Bedside Abdominal Ultrasound Findings as a Diagnostic and Prognostic Tool in Dengue Fever in Manipal Hospital, Bengaluru, India.* Cureus, 2024. **16**(7): p. e64734.

11. Ibrahim, M.A., et al., *The association of ultrasound assessment of gallbladder wall thickness with dengue fever severity.* Ultrasound J, 2022. **14**(1): p. 13.

12. Nainggolan, L., et al., *Gallbladder Wall Thickening for Early Detection of Plasma Leakage in Dengue Infected Adult Patients.* ACTA MEDICA INDONESIANA, 2018. **50**(3): p. 193-199.

13. Parmar, J.P., C. Mohan, and M. Vora, *Patterns of Gall Bladder Wall Thickening in Dengue Fever: A Mirror of the Severity of Disease.* ULTRASOUND INTERNATIONAL OPEN, 2017. **3**(2): p. E76-E81.

14. Parmar, J., et al., *"Honeycomb" pattern of gallbladder wall thickening - A forward step in early diagnosis of "Severe Dengue Fever".* INDIAN JOURNAL OF RADIOLOGY AND IMAGING, 2019. **29**(01): p. 14-18.
